# The Design and Feasibility of the: Radial Artery Puncture Hemostasis Evaluation – RAPHE Study, a Prospective, Randomized, Multicenter Clinical Trial

**DOI:** 10.3389/fcvm.2022.881266

**Published:** 2022-05-27

**Authors:** Péter Kulyassa, Balázs T. Németh, Réka Ehrenberger, Zoltán Ruzsa, Tibor Szük, Péter Fehérvári, Marie Anne Engh, Dávid Becker, Béla Merkely, István F. Édes

**Affiliations:** ^1^Heart and Vascular Center, Semmelweis University, Budapest, Hungary; ^2^Center for Translational Medicine, Semmelweis University, Budapest, Hungary; ^3^Invasive Cardiology Division, Department of Internal Medicine, University of Szeged, Szeged, Hungary; ^4^Department of Cardiology and Cardiac Surgery, University of Debrecen, Debrecen, Hungary; ^5^Department of Biomathematics and Informatics, University of Veterinary Medicine, Budapest, Hungary

**Keywords:** bleeding, hemostasis, radial artery occlusion, radial artery damage, radial approach angiography

## Abstract

**Introduction and Aim:**

Radial artery approach angiography is the current gold standard for coronary status diagnostics and eventual percutaneous revascularization (PCI). Currently, application of adequate, patent hemostasis based physical torniquets are used for puncture site control, to avoid bleeding, radial artery occlusion and damage (RAO and RAD). The Radial Artery Puncture Hemostasis Evaluation (RAPHE) is a prospective, randomized, multicenter clinical trial designed to investigate new, simplified techniques of radial artery hemostasis utilizing physical compression free methods.

**Methods and Results:**

The RAPHE study has been designed to evaluate the efficacy and safety of two non-compression based radial artery hemostasis methods: a 100% chitosan bioactive hemostatic dressing and a purpose-built radial potassium-ferrate based topical hemostasis disc. These devices will be investigated in a standalone configuration. Control group is a standard pneumatic airbladder-based compression device. A total of 600 patients will be enrolled in a three-way randomization (1:1:1) with two study and one control groups. Safety and efficacy endpoints are RAO, puncture site hematoma formation and RAD respectively, consisting of dissection, (pseudo)aneurism and/or fistula formation, measured post-procedure and at sixty days.

**Conclusion:**

The results from this trial will provide valuable information on new, simplified methods of radial artery hemostasis options and possibly simplify post-puncture management of patients.

**Clinical Trial Registration:**

[www.ClinicalTrials.gov], identifier [NCT04857385].

## Introduction

Transradial artery approach (TRA) coronary angiography and clinically driven percutaneous revascularization (PCI) has evolved into the worldwide standard in the past two decades. It has several benefits over the classical femoral approach including decreased mortality, less clinically significant bleeding, earlier patient mobilization, along with reduced hospital costs (1, 2) and is also preferred by most healthcare recipients. Radial artery use for increasingly complex coronary procedures have widened over the past years with even complex, high-risk rotational atherectomy based PCI successfully undertaken from a TRA, utilizing large bore guiding catheters. Bilateral use of the TRA has also been reported, in instances where additional entry points are required, as used in chronic total occlusion PCI. TRA is, however, not without its hindrances. Radial artery occlusion (RAO) and radial artery damage (RAD), encompassing vessel dissection, (pseudo)aneurism and eventual fistula formation are important associated phenomena accompanying TRA. Both findings were described in first ever publication reporting on TRA (3). Published meta-analysis research shows, that RAO frequencies vary on a large scale from < 1% all the way to 33% (4). Literature on the frequency and modality of RAD is less established, with vague data hinting at the fact that radial artery pseudoaneurysm and fistula formation occur seldom after TRA < 1% (5–8).

Furthermore, state-of-the-art hemostasis of a radial puncture site involves mechanical compression devices (MCD), (mainly using either a pneumatic balloon/airbladder or semiglobular compression rod) depending on various forms of so-called patent hemostasis (PH). To achieve this efficient and safe ipsilateral blood flow without puncture site bleeding a pulse oximeter is placed on the thumb of patients with simultaneous compression of the ulnar artery to eliminate retrograde flow through the palmar arches. After this torniquet pressure is gradually decreased and titrated until a signal on the oximeter is verified, demonstrating blood flow. This method assures, that blood flows though the punctured artery in an anterograde fashion yet does extravasate through the puncture site. This decrease in pressure must be performed every 10–20 minutes and always verified via the pulse oximeter. Streamlined PH methods have proven superior to fixed time, fixed pressure torniquet options (9–11). However, achieving any form of PH requires significant human resources and increased attention, especially during the initial 30 min after torniquet application. In higher volume catheterization laboratories one or more dedicated nurses must be allocated to ensure PH procedures are followed. If the required measures are not met to a full extent, that may compromise the patency procedure and lead to increased safety and efficacy concerns.

In recent years, development of non-compression based, hemostasis devices have emerged containing various, biological and/or chemical hemostatic elements. These achieve hemostasis without the need for long term pressure on the puncture sight. One of the two main products is the linear polysaccharide chitosan which acts as a bioactive, temporary seal to any bleeding wound (12, 13). Chitosan based bioactive techniques have been utilized in conjunction with MCDs to shorten torniquet times (14), leading to a significant decrease in pressure intervals (120 vs. 60/45/30 min). Also, in a recently published trial (15), standalone use of a catecholamine-chitosan based hemostatic device was compared to a pneumatic standard MCS. RAO rates were comparable, with 8.5 and 9.4% (*p* > 0.05) for the study and control device, respectively. Torniquet times were significantly shorter for the bioactive device (42 vs. 50 min, *p* < 0.01) with grade I and II bleeding frequencies also comparable (1.3 vs. 3.4%, *p* > 0.05). RAD rates were not reported.

Another device in clinical use incorporates a tailored hemostatic disc, with potassium-ferrate and a hydrophilic component as the active ingredients. The device rapidly dehydrates blood and causes prompt aggregation of proteins and cellular blood components, achieving quick hemostasis. Published data (16) underlines, that potassium-ferrate devices, when used in conjunction with a standard pneumatic MCD decrease hemostatic times (43 ± 14 min vs. 160 ± 43 min, *p* < 0.001), with no increase in bleeding complications (17.2 vs. 10.3%, *p* = 0.2) as compared to MCDs alone.

Standalone use (without mechanical augmentation) of these types of new bioactive hemostatic devices in TRA appears logical and could ease the required extensive manpower need of PH associated with MCDs and shorten compression times, yet sufficiently powered and purpose designed studies lack in this regard, which we seek to implement.

## Methods and Analysis

### Study Objectives and Definitions

The objective of the Radial Artery Puncture Hemostasis Evaluation (RAPHE) study is to evaluate the safety and efficacy of two novel, non-compression based, bioactive radial artery hemostasis devices: a 100% chitosan sponge bioactive dressing and the potassium-ferrate based topical discs applied in a standalone configuration. A pneumatic balloon inflatable MCD will serve as a well-established control.

RAPHE is a prospective, randomized, open-label, multicenter clinical trial, with three, high-volume university centers conducting the study, in Europe, Hungary.

### Endpoint Definitions and Hypothesis

The primary endpoint of the study will be a device oriented complex endpoint (DOCE). A complex endpoint has been defined, to assess hemostasis devices from both important clinical viewpoints: its efficacy in containing puncture site bleeding and its safety in terms of preserving radial artery patency and integrity.

DOCE will encompass the following: post-operative bleeding with visible hematoma formation after hemostasis device removal classified using the EASY methodology (17) (I), RAD, encompassing vessel dissection, (pseudo)aneurism and/or fistula formation (II) and RAO (III). These three parameters will be measured and quantified first at 24 hours after hemostasis device removal or at hospital discharge (whichever comes sooner), and again assessed at the 60-day follow-up visit.

Secondary endpoints will focus on human resource requirements and economics of devices. These include initial compression to achieve primary hemostasis (I), overall compression device usage time (II), and number of compression devices needed (III). Primary hemostasis is defined as the time in minutes, from application of the hemostasis device to the first successful decrease of the initial torniquet pressure, with success indicating no repeat bleeding from the puncture site. Overall compression time refers to total time of devices used as a hemostasis tool. In cases of acute bleeding following device use, a new, freshly opened tool may sometimes be required if the first one failed to contain bleeding. According to the RAPHE protocol, in case of initial device failure, a second, identical tool is to be utilized. If the second device also fails to contain puncture site bleeding, a bail-out hemostatic device will be employed. Thus, our last tertiary endpoint has been defined as follows: the need for a bailout compression method (I).

We hypothesize, that utilizing either of the novel bioactive devices in a standalone arrangement will prove clinically non-inferior to the other study and the control MCD, respectively. Furthermore, we assume, that the use of either one of the standalone bioactive devices will require less compression time, fewer human resources, and less management to successfully handle as compared to the MCD.

### Patient Enrollment, Inclusion/Exclusion Criteria

For potential enrollment in the RAPHE trial subjects must have a clinical indication for a radial approach coronary angiography and be between ages of 18 and 85 years. The use of standard 5, 6, or 7 French introducer sheaths will be required during the trial. A pre-screening sonographic evaluation of the radial artery will also be required to measure the diameter of the vessel. A minimum diameter of 1.8 mm-s is needed for potential enrollment. If a radial artery diameter of < 1.8 mm-s is detected, patients will discontinue the study as an early screen failure. Late screen-fail will occur if any other form of arterial puncture is needed during the procedure apart from the initial one, albeit upgrading the introducer size to a larger one will be allowed.

Exclusion criteria firstly focus on safety, thus potential patients shall be excluded based on the following: highly advanced age (>85 years) (I), sudden cardiac death and successful resuscitation prior to the index angiogram (II), unstable circulation prior to the index angiogram (III), pregnant or nursing (IV). Furthermore, three clinical exclusion criteria have been laid down, factors that are known to cause increased puncture site complications, to ensure no distortion of the collected data. These are: unusually small diameter of the radial artery (< 1.8 mm) (V), known and treated systemic autoimmune disease, e.g., rheumatoid arthritis, systemic lupus erythematosus etc. (VI) and severe, peripheral arterial vasculitis/disease, e.g., Buerger’s disease, Takayasu’s arteritis etc., (VII).

### Sample Size Calculation

Sample size calculation was carried out for non-inferiority studies following established guidelines (18, 19) with a significance level of 95% and a power of 80%. Although the best reported component of the DOCE, RAO rates range from < 1 to 33% (4), and it would be advisable to estimate sample size calculations for the worst-case scenario (i.e., 33%), however analyses of our own existing data regarding RAO and RAD (The RadPress trial—unpublished) deemed this value unreasonable for the centers involved. Therefore, we used a conservative 15% of the population rate for the DOCE for both intervention and control treatments. We allowed a 10% relative inferiority margin for the investigated treatments (20). The resulting sample size was 158 patients per arm. We also accounted for a potential 10% drop-out rate and factored in the uncertainty in true DOCE rates, and hence sample size estimates, and thus rounded the sample size to 200 patients per arm.

### Statistical Analyses

Data analysis will be done on an intention to treat basis. Baseline patient and disease characteristics will be summarized with detailed descriptive statistics that entail mean, median, SE, SD, IQR and range for continuous variables, while for categorical variables absolute and relative frequencies will be reported. Hypothesis testing of the primary outcome will be done with Fisher’s exact test with a significance level of 95%. Univariate analyses of secondary endpoints will be carried out as superiority analyses with one-way ANOVA, Kruskal-Wallis test, or logistic regressions, depending on the outcome variable type and distribution. Multivariate analyses of secondary endpoints will entail using general or generalized hierarchical mixed effects models depending on variable type and distribution. All statistical analyses will be done in the R statistical environment (21).

### Hemostatic Devices and Self-Adhesive Torniquet Wrap

The MCD that will be utilized in the RAPHE study has been in clinical use for more than 15 years, with established results (22). It features a single-use, transparent, purpose designed inflatable pneumatic airbladder (18 ml in volume) and a positioning marker for optimal placement at the junction of the introducer and intact skin. This MCD is held in place by a hook-and-loop fastener. Inflation of the device is achieved through a valve equipped line, using a custom made 20 ml syringe. This syringe allows for easy titration (0.5 ml increments) of air quantity within the pouch, allowing for effective PH measures. This MCD comes in two sizes (24 cm regular and 29 cm large) to accommodate for variable wrist sizes. It is used in catherization laboratories all over the world and thus makes for an excellent gold-standard device and as such the control MCD in the RAPHE study.

The first bioactive hemostatic device that will be investigated in study is a 100% chitosan sponge dressing. Chitosan is linear polysaccharide composed of randomly distributed deacetylated and acetylated D-glucosamine/N-acetyl-D-glucosamine units. It is produced from shells of shrimp and other crustaceans following alkaline treatment. It is a non-toxic, environmentally friendly substance with a variety of uses including agriculture, food supplementation and industrial applications. The one notable hindrance of the substance is potential allergic reactions, that although rare are known to manifest and thus inhibit its use in sensitive individuals. Chitosan may be formed and cut to any required shape and size. From a medical point of view, chitosan was first used in military practice, aiding bleeding control during battlefield operations (23). Civilian use was gradually introduced in the following years. The mechanism of action of chitosan is based on an electrical polarity difference between the positive charged seal and the negatively charged blood forming a strong mechanical barrier. Applying pressure to chitosan on a bleeding wound thus leads to rapid thrombocyte aggregation and thrombus formation. The RAPHE study will utilize a 3.5 × 3.5 cm sterile 100% chitosan sponge dressing that has been specifically developed to handle hemostasis of the radial and comparable size arteries.

The second bioactive device to be investigated will be a purpose designed topical disc containing potassium-ferrate powder and a hydrophilic agent as dual active ingredients. Potassium-ferrate is chemical compound with the formula K_2_FeO_4_. It is a highly reactive substance, that when encountering water, rapidly decomposes into ferric oxide (Fe_2_O_3_). The released cationic iron rapidly activates thrombocytes and initializes the coagulation cascade that leads to rapid thrombus formation. First medical application of this dual compound was in a powder form used to ease seeping hemorrhages around large bore catheter insertion sites. Eventually the technology evolved into a tailored disc containing a mix of compressed potassium-ferrate powder and a bioactive hydrophilic agent. This topical disc, when placed on a bleeding wound engages the hydrophilic active seal and desiccates the surrounding blood. As cationic-protonic exchange of potassium-ferrate decomposition takes place local pH levels are pushed to a highly acidic value, thereby again increasing blood desiccation. This process completes a strong hermetic seal.

Appropriate, quick, and effective fixation of the chitosan sponge dressing and potassium-ferrate discs are crucial for continues hemostasis control. We will be using the self-adherent elastic wraps to hold the bioactive devices in place. These do not require any further adhesives, pins, or clips to fixate and only bind to themselves. Using these novel wraps ensures excellent gripping of hemostatic devices and allow for fast un- and re-wrapping.

### Imaging Devices

Radial artery sonographic imaging is required multiple times during the trial and will be performed using either the Versana Active (GE Healthcare, Chicago, Illinois, United States) or EPIC 7 (Phillips Healthcare, Amsterdam, The Netherlands) complex ultrasound systems.

### Patient Data Collection

Collection of patient and procedure data will be performed using a web based electronic case report platform (eCRF).

Demographics that the RAPHE study will be gathering include age, gender, height, weight, and BMI. The accumulated patient history will focus on relevant cardiovascular and other diseases that are known or are assumed to influence hemostasis parameters and thus bleeding of study patients. Collected data will consist of presence of hypertension, presence of dyslipidemia, smoking habits, presence and type (insulin or non-insulin dependent) of diabetes mellitus, prior acute coronary events, prior PCI, prior surgical revascularization, prior stroke, presence of malignancy, presence of liver disease, left ventricular ejection fraction, glomerular filtration rate and thrombocyte count.

Data surrounding the radial puncture itself will also be collected in detail. Overall procedure time, radial artery diameter, any prior punctures, presence of calcium in the radial artery via sonographic evaluation, size of the largest applied introducer sheaths are all recorded.

All relevant times in conjunction with hemostasis device use are detailed and documented. Initial compression of potassium-ferrate and chitosan devices and initial air amount of the pneumatic MCD will be noted, along with the time needed to achieve primary hemostasis. Total time of hemostasis devices will be precisely measured up to the minute in accuracy.

Concomitant medication especially those, influencing puncture site bleeding episodes will all be precisely collected for later assessment. All parenteral and oral anti-coagulant and anti-platelet medication types and utilized doses will also be logged.

### Randomization Procedure

Randomization of consented subjects may only take place in the absence of exclusion criteria after removal of the last coronary catheter, by a physician participating in the study. It will take place in a 1:1:1 ratio, independently for every site via a computer algorithm, between the: pneumatic balloon MCD, potassium-ferrate disc and 100% chitosan sponge devices.

The pneumatic balloon based MCD serving as the control device will be placed on the introducer sheath inflated with the provided syringe to 12 ml-s with simultaneous removal of the introducer. An additional 2 ml-s of air will be inflated in case of acute bleeding and repeated if needed. It will be removed via a simplified patent hemostasis protocol (24), in which, after achieving primary hemostasis, exact titration to true PH we will be achieved by removing 2–3 ml-s of air every 10–15 min using a pulse oximeter to verify patency. At 0 ml-s of remaining air the device will be removed. These procedures will be carried out by qualified nursing staff. Nurses have been instructed to strictly adhere to the PH protocols and to contact study physicians in case of any potential adverse events, especially those in conjunction with radial hemostasis.

The standalone potassium ferrate disc and chitosan sponge devices will both be used in a similar way. They will be placed onto the junction of the intact skin and introducer and manually compressed. To aid better initial compression and prevent device adhesion to bloody surgical gloves, a ball gauze will be placed on the chitosan device. The potassium-ferrate disc will be compressed alone. During compression the introducer will be removed, allowing for a visible amount of blood to appear, which will activate the hemostatic properties in both devices. Strong manual compression will be held for 1 min. Devices will then be banded and held in place using self-adherent wraps as detailed above. Strong, occlusive fixation of the devices will be upheld for a total of 10 min, after which the self-adherent bond will be fully unwrapped until the hemostasis device is visible. By this time both devices will have activated and initiated the coagulation cascade and are firmly attached to the puncture site. If acute bleeding occurs, an additional, minimum 5 minutes of compression will be applied. If initial devices are no longer fit for use, a new similar one will be used. Re-wrapping will then occur without the application of relevant pressure, in a non-occlusive way. During this time the wrapping will only protect the hemostasis device from external influence (e.g., accidental dislodgement, slipping etc.). If the second randomized study device also fails to contain puncture site bleeding a bail-out device of choice shall be applied as per protocol, the choice of which is left to the discretion of the attending physician. After a period of 120 ± 15 min the self-adherent wrapping will be removed. The chitosan sponge device will also be removed using sterile saline irrigation. The potassium-ferrate disc will remain in place and removed by the patient after 24 hours post-procedure.

### Sonographic Evaluation and Follow-up

Examinations will be conducted three times during the trial, by a certified physician or licensed sonography specialist, blinded to all study procedures. The protocol will include a color and pulse-wave Doppler assessment and two-dimensional imaging in a longitudinal and transverse plane to assess arterial occlusion and/or damage, i.e., dissection, (pseudo)aneurism, and fistula formation.

The initial, pre-screening examination will be carried out prior to any puncture, to assess artery parameters and dimensions, accounting also for any potential intrinsic abnormalities of the region. The second sonographic evaluation of the puncture site will take place 24 hours after the procedure or at patient hospital discharge, whichever occurs first. All the above sonographic parameters will be collected, along with an Allen-test to account for palmar arch integrity along with radial artery bleeding and hematoma formation, which will be assessed using the EASY study classification. EASY class I and II will be considered minor, while III and above major bleeding events. Furthermore, all relevant clinical adverse events will also be collected and evaluated.

The third and final radial sonography and Allen-test will be conducted at 60 days follow-up, with a ± 10-day window. This time window was chosen as per empiric knowledge of the sites involved and also the fact that a previous trial (The RadPress trial—unpublished) with similar PH methods did not see any rational to further delay the follow-up visit to a later timepoint. Similarly, as with the first follow-up, all potential adverse events will be collected, and sonographic and clinical parameters of the radial puncture site examined.

### Flowchart


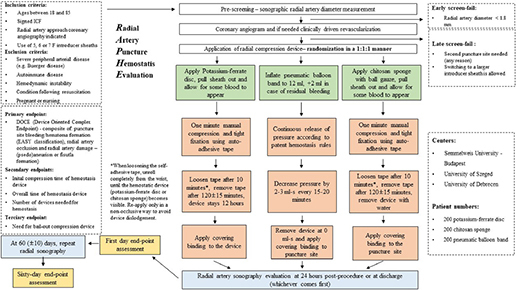


The above flowchart details the RAPHE trial algorithm of subject inclusion/exclusion, allocation, randomization, and clinical procedures. Specifics of all three arms are displayed along with the required post-operative and follow-up measures.

## Discussion

TRA has taken shape as the preferred approach to invasive coronary diagnostics and is also routinely used for all forms of contemporary PCI. The method is well established and favorable by most patients and healthcare providers, although important clinical hindrances are to be noted. The available data lead us to assume, that consideration toward preservation of radial artery integrity varies on quite large of a scale. Published meta-analysis data exhibits RAO ranges from < 1 to 33%, leading us to believe that many interventional establishments do not commit adequate resources and/or manpower to preserving the radial artery, while other institutions have dedicated personnel, post-operative observation rooms and in-house guidelines to maximize radial artery patency (4). As an important clinical part of DOCE, the established protocol of the RAPHE trial strongly focuses on preserving radial artery patency in all three arms. We expect RAO will be between 3 and 5% in all three arms of the RAPHE trial, with hopes of achieving a value of less than 3%.

Another component of DOCE, RAD is reported at surprisingly scarce frequencies. We strongly believe that this is due to the fact, that studies, incorporating detailed post-operative ultrasound evaluation are seldom, thus these phenomena appear massively underreported. With PH MCD use and non-compression based hemostasis devices we are of the opinion that RAD will definitely play a significant role in DOCE frequencies, maybe even occurring at a higher overall percentage than RAO. Thus, we expect an overall RAD rate (encompassing artery dissection, (pseudo)aneurism, and fistula formation) of between 5 and 8% with our rigorous sonographic follow-up evaluations.

Bleeding from the radial puncture site although less common as compared to femoral or brachial approaches still occurs at a clinically significant rate. Luckily, most bleeding issues do not require specific medical therapies, although they may be a cause for distress in patients especially when accompanied by discomfort and edema formation, which require topical care and pain alleviating medication. Serious, EASY III-V grade bleeding requiring specific, mostly surgical treatment occur rarely (< 1%) (17). Overall, clinical hematoma formation, assessable on the EASY scale (I-IV) is expected at around 5–7% in the RAPHE trial, with the majority (> 80%) of cases expected to be EASY class I and II.

RAPHE will focus on RAO, RAD and bleeding equally, from a device-oriented endpoint as all present clinical challenges influence quality of life of patients. With RAO, a conduit for further use is eliminated and clinical signs of upper extremity ischemia may present, necessitating surgical or endovascular treatments. Damage to the radial artery, clinically mostly manifesting as (pseudo)aneurysm and fistula formation can cause substantial pain/discomfort, paresthesia and even a palpable mass above the site of the puncture often causing a thrill in case of an arterio-venous fistula. These pathologies will likely require interventional methods or surgical repair to avoid chronic vascular conditions (5–8) and may cause a notable decrease in quality of life if left unchecked and/or untreated. Bleeding as the most common complication after TRA if serious may present as a significant or even critical issue. Thus a “perfect” compression device has a DOCE of zero per cent, as it causes no bleeding, RAO or RAD. In the RAPHE study we expect an overall DOCE rate, encompassing everything above of around 15% in all three arms of the trial.

The main goal of the study will be to determine, how standalone bioactive chitosan and/or potassium ferrate devices fare against the gold standard pneumatic airbladder based MCD. Besides clinically relevant safety and efficacy profiles, we will opt to also explore if fewer human resources are required to manage said bioactive devices. As per the RAPHE protocol, bioactive devices only require one adjustment, shortly after application and may require less overall compression time. Reducing the need for extensive human resource PH management and continuous MCD adjustments in high-volume centers is favorable, due to a multitude of factors. As bioactive devices do not require adjustments every 10–20 min, additional staff, managing strict PH protocols may be allocated elsewhere. In the RAPHE study we assume that patients randomized into one of the two investigational arms (potassium ferrate disc or chitosan sponge) will achieve primary hemostasis quicker and require less overall compression time as compared to MCD control subjects. From an economic point of view, the RAPHE trial will also be assessing the need of hemostasis units per patient in all three arms, and record if bail-out compression devices are needed. We have no convincing assumptions regarding these endpoints, due to a lack of prior reported data or in-house registries.

### Ethics and Dissemination

#### Ethical Considerations

The RAPHE clinical trial will be conducted in accordance with good clinical practice and the Helsinki Declaration (25). All regulatory and notified body requirements have been met to perform the study. The RAPHE study has been cataloged and authorized by the Hungarian National Institute of Pharmacy and Nutrition under number: OGYÉI/13123/2021. All enrolled patients will provide a written informed consent form, prior to any enrollment, study procedure or randomization, which will be collected by certified physicians participating in the study.

#### Safety and Termination

A Data Safety Monitoring Board (DSMB) consisting of three independent physicians who are not part of the RAPHE trial will perform an interim analysis after 50% of patients have been enrolled into a study. The DSMB will assess if any novel treatment option proves superior or inferior to the MCD used as the control. Statistical design will permit an early termination of the study if the DSMB identifies any potential advantages or disadvantages in any of the hemostasis options.

#### Data Management

All patient and procedure data will be stored on independent, encrypted, username and password protected servers with access restricted to study and DSMB personnel.

## Ethics Statement

The studies involving human participants were reviewed and approved by Hungarian National Institute of Pharmacy and Nutrition under number: OGYÉI/13123/2021. The patients/participants provided their written informed consent to participate in this study.

## Author Contributions

IÉ, PK, BN, and RE contributed to idea, conception, approval, and design of the study. PF provided all statistical analysis. IÉ wrote the initial draft. ME, PF, ZR, TS, DB, and BM wrote individual sections. All authors contributed to manuscript revision, read, and approved the submitted version.

## Conflict of Interest

The authors declare that the research was conducted in the absence of any commercial or financial relationships that could be construed as a potential conflict of interest.

## Publisher’s Note

All claims expressed in this article are solely those of the authors and do not necessarily represent those of their affiliated organizations, or those of the publisher, the editors and the reviewers. Any product that may be evaluated in this article, or claim that may be made by its manufacturer, is not guaranteed or endorsed by the publisher.
